# Supplementary material for: Tensile and Impact Toughness Properties of a Zr-Based Bulk Metallic Glass Fabricated via Laser Powder-Bed Fusion
Source: Materials (Basel). 2021 Sep 27;14(19):5627. doi: 10.3390/ma14195627 (PMC8510030; doi:10.3390/ma14195627)
Supplement: Supplementary file 1 [file materials-14-05627-s001.zip › materials-1377814-supplementary.pdf]

Article

Tensile and Impact Toughness Properties of a Zr-Based Bulk Metallic Glass Fabricated via Laser Powder-Bed Fusion

Navid Sohrabi [1,*], Annapaola Parrilli [2], Jamasp Jhabvala [1], Antonia Neels [2] and Roland E. Logé [1]

[1] Thermomechanical Metallurgy Laboratory, PX Group Chair, Ecole Polytechnique Fédérale de Lausanne (EPFL), 2002 Neuchâtel, Switzerland; jamasp.jhabvala@epfl.ch (J.J.); roland.loge@epfl.ch (R.E.L.)

[2] Center for X-ray Analytics, Swiss Federal Laboratories for Materials Science and Technology (Empa), Überlandstrasse 129, 8600 Dübendorf, Switzerland; annapaola.parrilli@empa.ch (A.P.); antonia.neels@empa.ch (A.N.)

* Correspondence: navid.sohrabi@epfl.ch

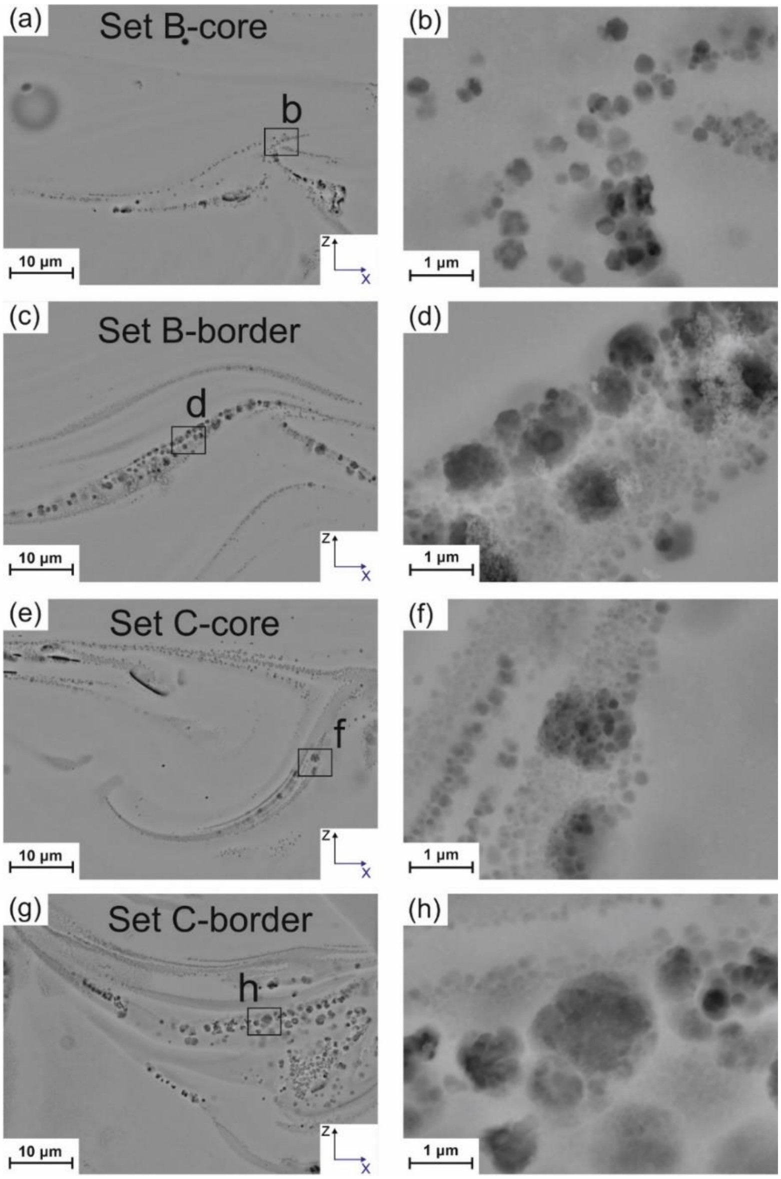

Figure S1. (**a**) BSE image of a melt pool in the core of Set B, (**b**) higher magnification of region b in (**a**), (**c**) BSE image of a melt pool in the border of Set B, (**d**) higher magnification of region d in (**c**), (**e**) BSE image of a melt pool in the core of Set C, (**f**) higher magnification of region f in (**e**), (**g**) BSE image of a melt pool in the border of Set C, and (h) higher magnification of region h in (**g**).

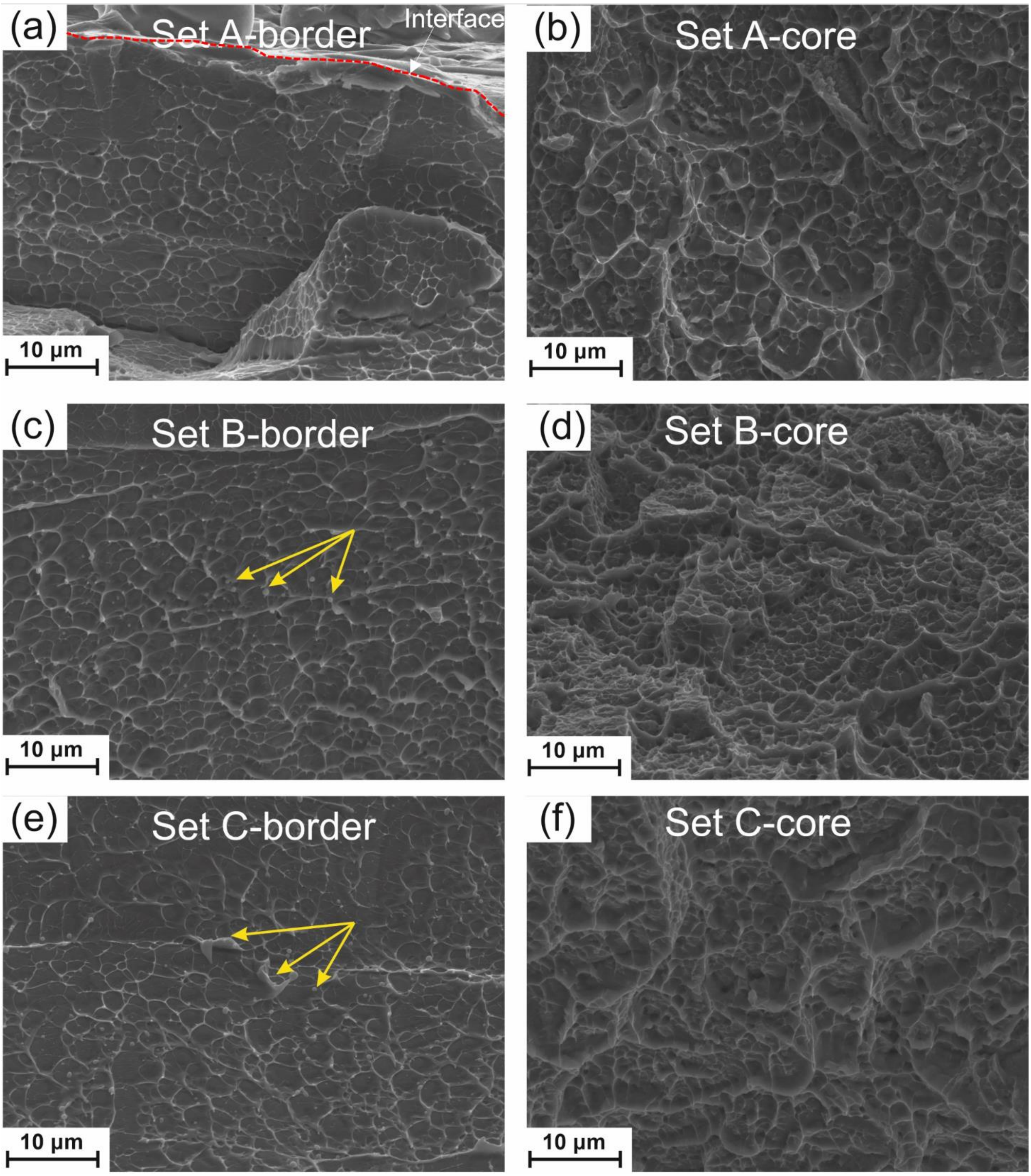

Figure S2. High magnification image of the fracture surface of the impact specimens fabricated via (**a**,**b**) Set A, (**c**,**d**) Set B, and (**e**,**f**) Set C. The red dashed line in (**a**) shows the interface of fractured surface and non-fractured region. The border region images (**a**,**c**,**e**) show vein-like patterns. For (**c**,**e**), which have higher impact toughness, molten particles are detected (yellow arrows). The images from the core region (**b**,**d**,**f**) are similar to each other, which shows that rapid fracture leads to similar fracture surface morphology. The images related to the core (**b**,**d**,**f**) were taken 2 mm away from the notch and into the core of the sample.

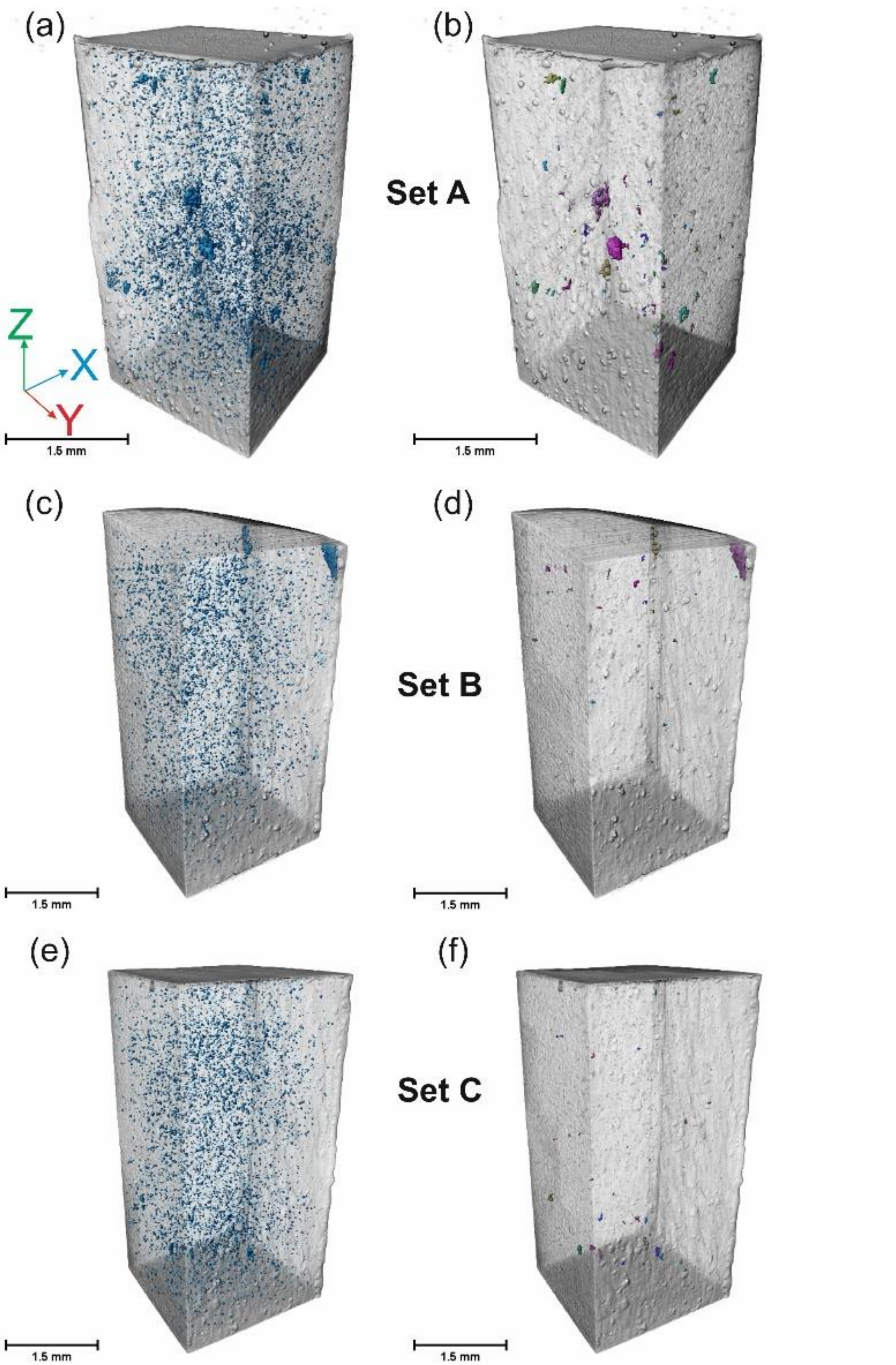

Figure S3. 3D constructed image of a part of tensile specimens fabricated via (**a**,**b**) Set A, (**c**,**d**) Set B, and (**e**,**f**) Set C using X-ray micro-computed tomography (Micro-CT). (**a**,**c**,**e**) include all defects, but (**b**,**d**,**f**) just shows LoFs.

Table S1. Comparison of impact toughness measured in the present study with other BMGs fabricated via conventional methods, such as casting and semi-solid forging.

Material	Impact toughness (J)	Size (mm³)	Comment	Method
AMZ4- Set A	0.123 ± 0.028	3 × 4 × 27	6% crystallization	Charpy
AMZ4- Set B	0.158 ± 0.019	3 × 4 × 27	9% crystallization	Charpy
AMZ4- Set C	0.163 ± 0.021	3 × 4 × 27	17% crystallization	Charpy
La BMG [6]	0.120	ϕ4×22	Amorphous	Izod
La BMG [6]	0.036	ϕ4×22	30% crystallization	Izod
La BMG [7]	0.700	2.6 × 5 × 22	Amorphous	Izod
La BMG [7]	0.170	2.6 × 5 × 22	Amorphous-annealed	Izod
Vitreloy 1 [8]	0.950	3 × 5 × 30	Amorphous	Charpy
Vitreloy 1 [8]	0.050	3 × 5 × 30	Amorphous-annealed	Charpy
Vitreloy 1 [9]	1.150	3 × 6 × 30	Amorphous	Charpy
Vitreloy 1 [9]	0.100	3 × 6 × 30	Amorphous-annealed	Charpy
Vitreloy 1 [10]	1.200	3 × 6 × 30	Amorphous	Charpy
Vitreloy 1 [10]	0.180	3 × 6 × 30	Amorphous-annealed	Charpy
Vitreloy 1 [11]	0.680	3 × 3 × 30	Amorphous	Charpy
LM2 [11]	0.130	3 × 3 × 30	40% crystallization*	Charpy
DV1 [11]**	4.820	3 × 3 × 30	40% crystallization*	Charpy
DH3 [11] **	5.27	3 × 3 × 30	67% crystallization*	Charpy
DH3 [11]	0.190	3 × 3 × 30	100% crystallization*	Charpy
Ti-6Al-4V [11]	2.97	3 × 3 × 30	Crystalline	Charpy
Al-7075 [11]	0.38	3 × 3 × 30	Crystalline	Charpy
Al-2024 [11]	0.66	3 × 3 × 30	Crystalline	Charpy
DH3 [12]	0.820	3 × 3 × 30	Amorphous	Charpy
DH3 [12]	2.660	3 × 3 × 30	67% crystallization*	Charpy
DV1 [12]	1.310	3 × 3 × 30	40% crystallization*	Charpy

* Ductile crystalline phase

**Semi-solid forging
